# Levels of Salmonella enterica and Listeria monocytogenes in Alternative Irrigation Water Vary Based on Water Source on the Eastern Shore of Maryland

**DOI:** 10.1128/Spectrum.00669-21

**Published:** 2021-10-06

**Authors:** Chanelle L. Acheamfour, Salina Parveen, Fawzy Hashem, Manan Sharma, Megan E. Gerdes, Eric B. May, Koriante Rogers, Joseph Haymaker, Rico Duncan, Derek Foust, Maryam Taabodi, Eric T. Handy, Cheryl East, Rhodel Bradshaw, Seongyun Kim, Shirley A. Micallef, Mary Theresa Callahan, Sarah Allard, Brienna Anderson-Coughlin, Shani Craighead, Samantha Gartley, Adam Vanore, Kalmia E. Kniel, Sultana Solaiman, Anthony Bui, Rianna Murray, Hillary A. Craddock, Prachi Kulkarni, Rachel E. Rosenberg Goldstein, Amy R. Sapkota

**Affiliations:** a Department of Agriculture, Food, and Resource Sciences, University of Maryland Eastern Shore, Princess Anne, Maryland, USA; b United States Department of Agriculture, Agricultural Research Service, Beltsville Agricultural Research Center, Environmental Microbial and Food Safety Laboratory, Beltsville, Maryland, USA; c Department of Epidemiology and Biostatistics, University of Maryland School of Public Health, College Park, Maryland, USA; d Department of Natural Sciences, University of Maryland Eastern Shore, Princess Anne, Maryland, USA; e Department of Plant Science and Landscape Architecture, University of Maryland, College Park, Maryland, USA; f Centre for Food Safety and Security Systems, University of Maryland, College Park, Maryland, USA; g Maryland Institute for Applied Environmental Health, University of Maryland School of Public Health, College Park, Maryland, USA; h Department of Animal and Food Sciences, University of Delaware, Newark, Delaware, USA; University of Minnesota

**Keywords:** *Salmonella*, *Listeria monocytogenes*, irrigation, filtration, modified Moore swab, mid-Atlantic, reclaimed water

## Abstract

Irrigation water sources have been shown to harbor foodborne pathogens and could contribute to the outbreak of foodborne illness related to consumption of contaminated produce. Determining the probability of and the degree to which these irrigation water sources contain these pathogens is paramount. The purpose of this study was to determine the prevalence of Salmonella enterica and Listeria monocytogenes in alternative irrigation water sources. Water samples (*n* = 188) were collected over 2 years (2016 to 2018) from 2 reclaimed water plants, 3 nontidal freshwater rivers, and 1 tidal brackish river on Maryland’s Eastern Shore (ESM). Samples were collected by filtration using modified Moore swabs (MMS) and analyzed by culture methods. Pathogen levels were quantified using a modified most probable number (MPN) procedure with three different volumes (10 liters, 1 liter, and 0.1 liter). Overall, 65% (122/188) and 40% (76/188) of water samples were positive for S. enterica and L. monocytogenes, respectively. For both pathogens, MPN values ranged from 0.015 to 11 MPN/liter. Pathogen levels (MPN/liter) were significantly (*P < *0.05) greater for the nontidal freshwater river sites and the tidal brackish river site than the reclaimed water sites. L. monocytogenes levels in water varied based on season. Detection of S. enterica was more likely with 10-liter filtration compared to 0.1-liter filtration. The physicochemical factors measured attributed only 6.4% of the constrained variance to the levels of both pathogens. This study shows clear variations in S. enterica and L. monocytogenes levels in irrigation water sources on ESM.

**IMPORTANCE** In the last several decades, Maryland’s Eastern Shore has seen significant declines in groundwater levels. While this area is not currently experiencing drought conditions or water scarcity, this research represents a proactive approach. Efforts, to investigate the levels of pathogenic bacteria and the microbial quality of alternative irrigation water are important for sustainable irrigation practices into the future. This research will be used to determine the suitability of alternative irrigation water sources for use in fresh produce irrigation to conserve groundwater.

## INTRODUCTION

Enhanced awareness of the health benefits of fresh produce led to an average 4.5% per year increase in the consumption of fruits and vegetables worldwide in 1990 to 2004 (1, 2). While considered healthy, this increase in fresh produce consumption does not come without risks to public health due in part to the fact that these food products are usually consumed raw or with minimum processing (3). Consequently, as consumption of fresh produce has risen, outbreaks of foodborne illnesses associated with these foods have risen as well. According to the Centers for Disease Control and Prevention (CDC), 46% of all foodborne illnesses and 23% of foodborne illness related deaths in the United States annually can be attributed to ingestion of contaminated produce (4). While determining the source of produce-associated outbreaks can be difficult, produce contamination can occur at any point from farm to fork. One suspected route of contaminated produce is irrigation water (5–7). This research focuses on irrigation water sources on the Eastern Shore of Maryland (ESM), located in the mid-Atlantic region of the United States. ESM is an agriculturally intensive area, generating over 70% and 44% of Maryland’s total agricultural and fresh produce sales, respectively, in 2017 (8).

Globally, the agriculture sector is the largest consumer of available freshwater, responsible for approximately 70% of total freshwater withdrawals (9). However, climate change and water scarcity are placing severe stress on available freshwater irrigation water sources, especially in drought-stricken areas (10). Declines in annual precipitation in the western United States are expected to reduce water supplies for irrigation during the summer and fall growing seasons (11). While the mid-Atlantic is currently not experiencing water scarcity, this is predicted to change in the coming years (12–14). In this region, groundwater is the most commonly used water source for agricultural irrigation, accounting for 53% of irrigation water withdrawals (14, 15), and increased demand has led to a steady decline in groundwater levels in this region (14). In 2005, it was reported that the Coastal Plain region of Maryland, including ESM, had a 2-foot per year decline in groundwater levels between 1980 and 2005, potentially affecting the long-term sustainability of groundwater resources in this area (12). Moreover, ESM is estimated to be at moderate risk for decreases in irrigation water supplies due to drought in the future (13). With the demand for water in the agriculture sector expected to increase by 2050 and the continued depletion of groundwater-fed aquifers, safe use of alternative water sources for fresh produce irrigation has become an area of great interest (16, 17).

There have been a number of studies showing variations in the microbial quality of irrigation water sources in the mid-Atlantic region. Micallef et al. (18) found 7.7% (*n* = 39) and 15% (*n* = 13) of pond water samples and irrigation ditch water samples to be positive for Salmonella on mid-Atlantic tomato farms in 2009 and 2010, respectively. Weller and associates (19) found that 12% (*n* = 10) and 6% (*n* = 4) of irrigation water samples were positive for L. monocytogenes and Salmonella, respectively, collected from 10 farms in New York state over a 6-week period. These authors also found that 63% and 6% of irrigation water samples in 2014 (19) and 2017 (20), respectively, from the same site in New York were positive for L. monocytogenes. Truitt and coworkers (21) found an overall prevalence of Salmonella of 19% (*n* = 400) in preharvest production waters on the Eastern Shore of Virginia. Of surface river waters sampled on ESM, 65% were positive for S. enterica in 2015 and 2016 (6). More recently, Sharma et al. (22) found that 50% and 31% of nontraditional irrigation water sources in the mid-Atlantic tested positive for S. enterica and L. monocytogenes, respectively.

The study presented here utilizes the same methodology as our previous research (22) but with different sampling sites in different geographic areas. In that study, the irrigation water sites sampled were mainly on the Western Shore of Maryland (WSM). While in the same region, these two geographic areas are very distinct from each other in many regards, including climate, agricultural land use, topography, soil composition, and population. It has been suggested that different environmental factors in different regions may affect Salmonella prevalence (5). As seen in the Weller studies previously mentioned (19, 20), variation can be seen even within the same site at different points in time. Thus, the current study investigates whether there may be variations the prevalence of these two pathogens in an area with different geography, land use, and water types from our previous study.

In addition, this research uses a subset of sites used in our previous research (23). That previous study reported a low prevalence (2.35%) of Shiga-toxigenic Escherichia coli in these water sources. The focus of the current research is the two pathogenic bacteria that rank in the top five in terms of the economic burden they place on public health in the United States, Salmonella enterica and Listeria monocytogenes (24). From 2010 to 2017, 85 multistate outbreaks associated with fresh produce occurred in the United States (25). Of these 85, 56 were attributable to Salmonella, resulting in 3,778 illnesses, 778 hospitalizations, and 16 deaths; 4 were attributable to L. monocytogenes, leading to 173 illnesses, 169 hospitalizations, and 37 deaths (25). The overall goal of this study was to determine the microbiological quality, with respect to the prevalence of S. enterica and L. monocytogenes, in alternative irrigation water sources (26) on ESM.

## RESULTS

### Recovery of pathogens from different sites.

**FIG 1:**
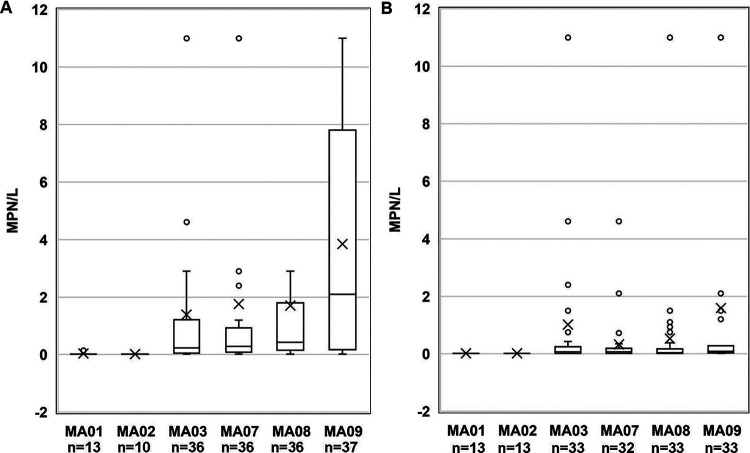
(A and B) Bacterial prevalence in MPN/liter for (A) S. enterica and (B) L. monocytogenes by site (*n* = number of samples) on the Eastern Shore of Maryland from October 2016 to October 2018. The boxplots show the median and the 25th and 75th percentiles of the range. The whiskers show observations that are lower and higher than the 25th and 75th percentiles, respectively.

Overall, S. enterica and L. monocytogenes were recovered from 65% (122/188) and 40% (76/188) of sampling events, respectively. Table 1 shows the number of sampling events where each water volume filtered contained S. enterica or L. monocytogenes. Levels of S. enterica and L. monocytogenes, measured as most probable number (MPN/liter), varied among sites over the course of the study. S. enterica MPN/liter values for the nontidal freshwater river sites MA03 (1.20 MPN/liter), MA07 (1.96 MPN/liter), and MA09 (4.09 MPN/liter) and the tidal brackish river site MA08 (2.18 MPN/liter) were significantly (*P < *0.05) greater than those for the reclaimed water sites MA01 (0.04 MPN/liter) and MA02 (0.02 MPN/liter) (Fig. 1). Within nontidal freshwater river sites, S. enterica levels were also significantly (*P = *0.005) higher for MA09 than for MA03. Similarly, L. monocytogenes MPN/liter values were significantly (*P < *0.05) greater for MA03 (1.12 MPN/liter), MA07 (0.32 MPN/liter), MA09 (1.55 MPN/liter), and MA08 (0.53 MPN/liter) than for MA01 (0.015 MPN/liter) and MA02 (0.015 MPN/liter) (Fig. 1). Within nontidal freshwater river sites, L. monocytogenes levels were significantly (*P = *0.022) greater for MA09 than MA07.

**TABLE 1:** Number of sampling events where each water volume filtered contained S. enterica or L. monocytogenes

Site	Water type	No. of sampling events	Data for [no. (%)]:					
			S. enterica			L. monocytogenes		
			0.1 liter	1 liter	10 liters	0.1 liter	1 liter	10 liters
MA01	Reclaimed water	23	2 (8.7)	1 (4.3)	2 (8.7)	0	0	0
MA02	Reclaimed water	18	0	0	1 (5.6)	0	0	0
MA03	Nontidal freshwater	36	15 (41.7)	22 (61.1)	25 (69.4)	12 (33.3)	13 (36.1)	17 (47.2)
MA07	Nontidal freshwater	36	13 (36.1)	22 (61.1)	31 (86.1)	17 (47.2)	15 (41.7)	20 (55.6)
MA08	Tidal brackish water	37	22 (59.5)	28 (75.7)	30 (81.1)	11 (29.7)	13 (35.1)	20 (54.1)
MA09	Nontidal freshwater	38	24 (63.2)	28 (73.7)	33 (86.8)	17 (44.7)	18 (47.4)	19 (50)

### Water type and seasonality affected recovery of pathogens.

**FIG 2:**
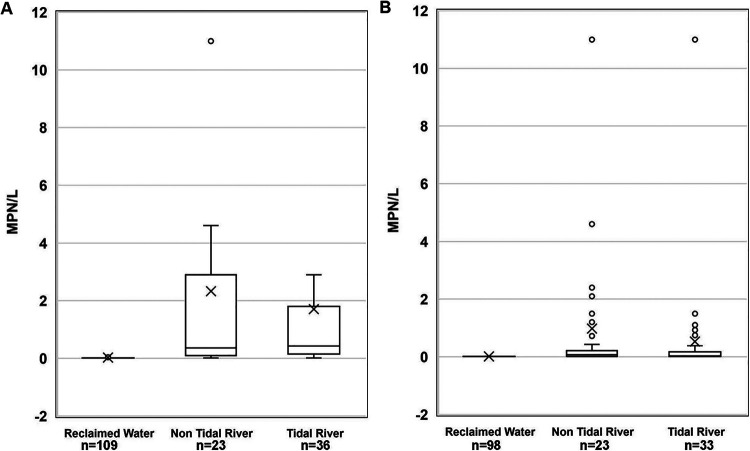
(A and B) Bacterial prevalence in MPN/liter for (A) S. enterica and (B) L. monocytogenes by water type (*n* = number of samples) on the Eastern Shore of Maryland from October 2016 to October 2018. The boxplots show the median and the 25th and 75th percentiles of the range. The whiskers show observations that are lower and higher than the 25th and 75th percentiles, respectively.

**FIG 3:**
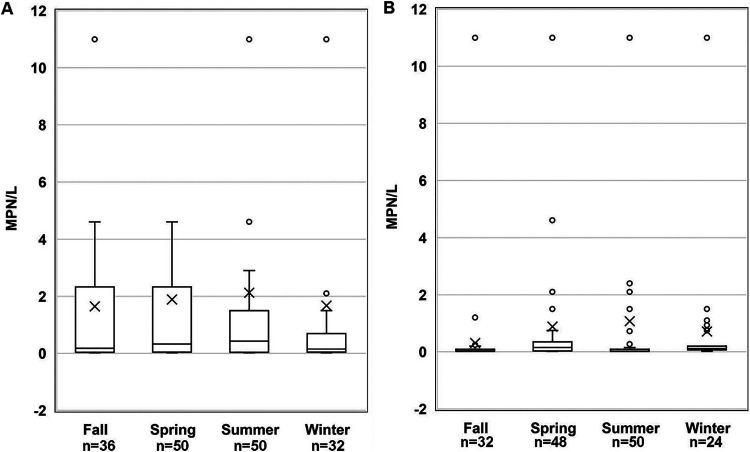
(A and B) Seasonal variation in bacterial prevalence in MPN/liter for (A) S. enterica and (B) L. monocytogenes (*n* = number of samples) on the Eastern Shore of Maryland from October 2016 to October 2018. The boxplots show the median and the 25th and 75th percentiles of the range. The whiskers show observations that are lower and higher than the 25th and 75th percentiles, respectively.

When compared by water type, S. enterica and L. monocytogenes levels were significantly (*P < *0.05) lower in reclaimed water than in nontidal freshwater and tidal brackish water (Fig. 2). No significant (*P > *0.05) differences were detected in pathogen levels between the nontidal freshwater and tidal brackish water sites. Seasonality did not affect S. enterica levels, with statistically similar (*P > *0.05) levels found in all seasons (Fig. 3). In contrast, L. monocytogenes levels were significantly higher in spring and winter (*P = *0.0069 and *P = *0.0304, respectively) than in fall (Fig. 3).

### Volume of water filtered affected the likelihood of Salmonella enterica recovery.

Table 2 shows the odds ratios for recovery of S. enterica and L. monocytogenes by volume filtered. For S. enterica, the odds of recovery (2.15) were significantly (*P = *0.0129) higher when 10 liters of water was filtered than when 0.1 liter was filtered. Filtering 1 liter of water had 1.82 times the odds of recovering S. enterica compared to filtering 0.1 liter of water, and filtering 10 liters of water had 2.15 times the odds of recovering S. enterica compared to filtering 0.1 liter of water. For L. monocytogenes, there were no statistically significant differences in the odds of recovery by volume of water filtered.

**TABLE 2:** Odds ratio (OR) estimates for S. enterica and L. monocytogenes recovery^*a*^

Pathogen	Filtered vols compared	OR estimate	*P* value
S. enterica	1 liter vs 0.1 liter	1.82	0.0572
	10 liters vs 0.1 liter	2.15	0.0129
	10 liters vs 1 liter	1.18	0.5996
L. monocytogenes	1 liter vs 0.1 liters	0.68	0.1880
	10 liters vs 0.1 liter	0.71	0.2108
	10 liters vs 1 liter	1.04	0.9000

a Only samples where either S. enterica or L. monocytogenes were recovered were used.

### Ordinal relationships between the pathogens and environmental characteristics.

**FIG 4:**
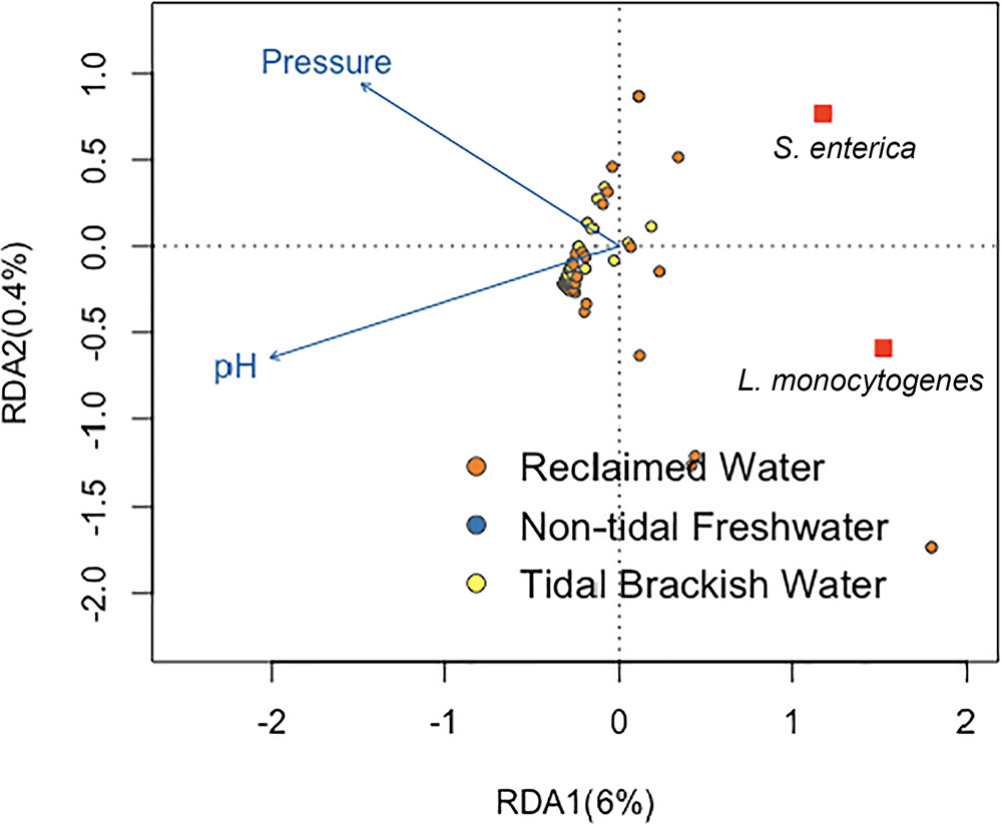
The total constrained variance (6.4%) is plotted on the *x* axis (RDA 1 [6%]) and *y* axis (RDA 2 [0.4%]). (*n* = 117) The direction of increase (increasing value of the parameter) is indicated by the direction the blue arrows in the RDA plot are pointing. These arrows are pointing away from the pathogens (red squares), indicating a negative association between these parameters and the two pathogens. Shorter distances between dots (S. enterica, L. monocytogenes, water types) and water quality parameters (blue arrows) indicate higher levels of that characteristic relative to categorical (water types) or response (pathogen) variables.

The ordinal relationships between S. enterica and L. monocytogenes and the 10 environmental characteristics (water type, conductivity, salinity, oxygen-reducing potential, dissolved oxygen, turbidity, nitrate, temperature, barometric pressure, and pH) tested are shown in the redundancy analysis (RDA) plot (Fig. 4). An analysis of variance using permutation was used to determine the significance of the constraining variables (environmental characteristics) and categorical values (water types) in the RDA model. Out of the environmental characteristics tested, pH and barometric pressure were determined to be significant (*P = *0.008 and *P = *0.049, respectively) factors in relation to S. enterica and L. monocytogenes levels. Both pathogens were negatively associated with increasing barometric pressure and pH (blue arrows are pointing away from the pathogen points [red squares]). Figure 4 shows that while most of the water samples are clustered around the center of the RDA ordination graph, several reclaimed water samples had a positive association with both pathogens, pointing to further investigation of both pathogens in reclaimed water compared to other water types. The total constrained variance accounted for by the water physicochemical factors in the RDA plot (6.4%) is represented by the eigenvectors RDA1 (*x* axis) and RDA2 (*y* axis).

## DISCUSSION

ESM is currently experiencing decline in groundwater levels, and this is predicted to worsen in the future. In an effort to conserve groundwater, exploration of alternative water sources for irrigation has become a key funding priority of the U.S. Department of Agriculture (USDA) National Institute of Food and Agriculture (27). With groundwater being the primary water type used for irrigation on ESM, alternative water sources in this region include reclaimed water, untreated surface water, and brackish water (26).

The results of this study demonstrated levels of S. enterica and L. monocytogenes much lower in the reclaimed water samples than in the nontidal freshwater rivers and the tidal brackish river water samples. These data are consistent with our previous research (22). Since the reclaimed water was tested after undergoing primary, secondary, and tertiary treatments to remove pathogens and other contaminants prior to lagoon storage, lower pathogen levels were expected. The use of reclaimed water for agricultural irrigation may offer several advantages: (i) it constitutes a reliable and renewable water source that is climate-resilient, (ii) it can be an affordable source of irrigation water, and (iii) its use would alleviate the burden on ground water sources (27, 28).

The prevalence rates of S. enterica (65%) and L. monocytogenes (40%) in the current study were similar to those from Cooley et al. (5), who found S. enterica and L. monocytogenes prevalence to be 65% and 43%, respectively, in agricultural watersheds in central California. However, the prevalence levels in the current study were greater than those reported in our previous research for distinct sites located elsewhere (22), where the occurrence of S. enterica and L. monocytogenes was 50% and 31%, respectively, using the same sampling protocol and sampling dates. In that study, the prevalence of both S. enterica and L. monocytogenes was lower in reclaimed waters and in ponds than in rivers. For the current research, sampling sites were all located on ESM. This area is densely populated with poultry operations. All of the surface water sites sampled were within 4 km of several operational poultry facilities (Table 3 [29]). In addition, MA03 was within 1.6 km of a wastewater treatment discharge facility. Previous studies have shown that surface waters close to confined poultry feeding operations are susceptible to pathogen contamination (30, 31). Vereen et al. (32) found that Salmonella detection frequencies were positively associated with the number of poultry houses in subwatersheds in Georgia and found that Salmonella prevalence increased near wastewater treatment plant discharge points (32). Moreover, runoff or dust from poultry farms or from field poultry litter application (6) may have contributed to the higher incidence of these pathogens found in these water sources.

**TABLE 3:** Description of sites and number of samples taken by season, fall 2016 to 2018

Site	Description	Data for [no. (%)]:			
		Spring	Summer	Fall	Winter
MA01	Reclaimed. Influent treatment: activated sludge processing (sequential batch reactor), filtration, UV light, and chlorination. Storage: open-air lagoon before land application. Sample collection: spigot in the irrigation line of sprinkler heads.	5 (21.7)	8 (34.8)	10 (43.5)	N/A^*a*^
MA02	Reclaimed. Influent treatment: activated sludge processing (sequential batch reactor), filtration, UV light, and chlorination. Storage: open-air lagoon before land application. Sample collection: spigot at the base of the center pivot.	3 (16.7)	8 (44.4)	7 (38.9)	N/A^*a*^
MA03	Nontidal freshwater creek, tributary of the Nanticoke River. Catchment area: width, ⁓3 m and depth ⁓1 m; wooded, agronomic cropland adjacent to the creek (⁓30–50 m); within 1.6 km downstream from wastewater treatment discharge facility and one operational poultry facility.	7 (19.4)	11 (30.6)	11 (30.6)	7 (19.4)
MA07	Nontidal freshwater creek, tributary of the Nanticoke River. Catchment area: width, ⁓10 m and depth, ⁓1 m; flood plain grasses and woodland (hardwoods); within 4 km downstream from several operational poultry facilities.	7 (19.4)	11 (30.6)	11 (30.6)	7 (19.4)
MA08	Tidal brackish river flowing into the Chesapeake Bay. Catchment area: width, ⁓15 m and depth, ⁓2–3 m; marsh grasses on both sides (⁓25–50 m wide), then pine woods; within 1.5–2.5 km downstream from several operational poultry facilities.	7 (18.9)	11 (29.7)	12 (32.4)	7 (18.9)
MA09	Nontidal freshwater creek, tributary of the Pocomoke River. Catchment area: width, ⁓8 m and depth, ⁓1 m; forested and agronomic cropland; located less than 1.5 km downstream from several poultry facilities.	7 (18.4)	11 (28.9)	13 (34.2)	7 (34.2)

a N/A, not applicable. No samples were collected from MA01 and MA02 in winter due to inclement weather. Adapted from Solaiman et al. (29).

S. enterica and L. monocytogenes incidence in rivers was greater in this study than in other studies evaluating the presence of these pathogens in pond surface waters in this region. Micallef et al. (18) found 7.7% (*n* = 39) of pond water samples were positive for Salmonella. More recently, Truitt et al. (21) found the overall prevalence of Salmonella from 20 agricultural ponds on the Eastern Shore of Virginia in 2015 and 2016 to be 19% (*n* = 400). This variation in prevalence between microbial quality of river and pond surface water was also reported for indicator bacteria (29) and for creek versus irrigation pond sites (33) and is of critical importance for food safety. Moreover, other factors may confound the data and make direct comparisons more challenging. This may include year to year variation and season of collection (33), as well as sample volumes analyzed. The current research evaluated three sample volumes (0.1 liters, 1 liter, and 10 liters). Filtering 1 liter and 10 liters was 1.82 and 2.15 times more likely to recover S. enterica than filtering 0.1 liters, respectively. Our previous research (22) showed that filtering 10 liters was 43.5 and 25.5 times more likely to recover S. enterica than filtering 1 liter and 0.1 liter, respectively. The relatively small increase in the likelihood of recovering S. enterica when filtering 10 liters versus 1 liter of water from these sites in the present study compared to the sites in our previous research indicates the greater prevalence of S. enterica in the specific river sources we evaluated. These results indicate that both the volume filtered and the volume analyzed can affect the recovery of S. enterica and L. monocytogenes.

The lack of a seasonal effect on S. enterica prevalence is in agreement with other studies (3, 34). However, significantly higher levels of Salmonella spp. are frequently reported during the summer compared to the other seasons (33). L. monocytogenes populations in water were significantly greater in winter and spring than fall in the current study. These results are consistent with Cooley et al. (5), whose results also showed greater L. monocytogenes prevalence in winter and spring than fall. Similarly, several investigators have found that L. monocytogenes was more frequently detected in waters with colder temperatures (3, 22).

The results of the RDA analysis show that little variance in S. enterica and L. monocytogenes levels was attributable to the water physicochemical factors measured, with only 6.4% of the variance constrained in the RDA. This finding is consistent with other studies (22, 35). McEgan et al. (35) found weak linear relationships between Salmonella levels and water physicochemical indicators in surface waters in central Florida; however, they did find site-specific correlations in some cases. Sharma et al. (22) reported poor correlations between water physiochemical factors and S. enterica and L. monocytogenes, with a total constrained variance of 13.8%. Weller et al. (20) found that interactions between environmental factors affected the likelihood of pathogen detection in agricultural waters in Arizona and New York. These authors concluded that the food safety risks associated with use of a water source for irrigation are dependent on environmental conditions at the time of water use (20). The variability in these results emphasizes the regionality of microbial water quality characteristics. Therefore, it is not redundant to investigate how local environmental conditions affect the survival and persistence of pathogens in irrigation water and what indicators of microbial quality to use for various irrigation water types.

This study shows that S. enterica and L. monocytogenes are present in at high levels in alternative irrigation water sources on ESM. Several produce-related foodborne illness outbreaks have been traced back to this agricultural area (36, 37). From a microbial perspective, due to lower pathogen loads, reclaimed water may offer an affordable and sustainable agricultural irrigation water source in this region. However, due to the small sample sized used in this study, further research is needed. The difference in incidence of positive samples compared to previous studies in other geographic areas stresses the need for regional studies that evaluate the unique conditions and environmental prevalence of foodborne pathogens in irrigation water sources. This research can be used to determine the most suitable alternative water sources for use in fresh produce irrigation in this region, combining food safety and conservation goals. Moreover, the results of this research will help identify water sources that require on-farm mitigation strategies to reduce bacterial populations and improve the quality of those water sources for irrigation.

## MATERIALS AND METHODS

### Experimental sites.

Water samples were collected over 2 years (October 2016 to October 2018) from six sites (four surface water and two reclaimed water sites) on Maryland’s Eastern Shore. The four surface water sites which included: three nontidal freshwater rivers (MA03, *n* = 36; MA07, *n* = 36; MA09, *n* = 37) and one tidal/brackish river (MA08, *n* = 36), were sampled twice monthly during the growing season (October 2016, June-October 2017, May-October 2018) and once a month during the off season (November 2016-May 2017, November 2017-April 2018). The reclaimed irrigation water sources (MA01, *n* = 13; MA02, *n* = 10) were only in use during the growing season; therefore, sampling at these sites was conducted from April 2017 to October 2018 (Table 3; 29).

### Modified Moore swab design.

On-site water collection was conducted using MMS adapted from Sbodio et al. (38) and modified in previous research (22, 23). MMS were created using grade no. 90 cheesecloth (Lion Services, Inc., Charlotte, NC, USA) folded in thirds lengthwise and rolled into a cylindrical shape 16 cm long and 4 cm in diameter. Autoclaved MMS was aseptically inserted in a 29.2-cm long and 3.81-cm diameter polyvinyl chloride (PVC) cartridge. The PVC cartridge was designed with a mesh screen on one side for water inlet and debris filtration and a 3.81-cm male barbed PVC fitting on the opposite end for connection to a water pump.

### Water sample collection.

This study utilized a modified MPN approach using three volumes (0.1 liters, 1 liter, and 10 liters) collected in triplicate (22). For the 0.1- and 1-liter samples, water was collected in a sterile 1-liter bottle, transferred to a sterile graduated cylinder, and measured to the target volume. The target volume of water was then poured through the mesh screen of a sterile cartridge containing an MMS and allowed to flow through by gravity filtration. For the 10-liter samples, the cartridge was inserted into a PVC flotation device allowing for collection of samples from a depth of 15 cm to minimize sediment disturbance. The cartridge was connected to a water pump (Honda model no. WX10TA; Honda Power Equipment, Alpharetta, GA, USA), and 10 liters of water was actively pumped through the MMS. At the reclaimed water sites, 20-liter water samples were collected from spigots near field discharge points into a sterile 20-liter carboy and treated with 20 ml of 10% sodium thiosulfate (Fisher Scientific, Washington, DC, USA) to neutralize any free chlorine in the water. Water was then filtered as described above. After filtration, MMS were placed in labeled Whirl-Pak bags (Nasco, Madison, WI, USA) and transported on ice to the laboratory.

At each sample collection site, the water physicochemical parameters water temperature (°C), dissolved oxygen (mg/liter), conductivity (μS/cm), pH, oxygen reducing potential (mV), salinity (PNU), and turbidity (FNU) were also measured using an EXO2 multiparameter water quality sonde (YSI, Yellow Springs, OH, USA). Precipitation amounts (1, 7, and 14 days prior to sampling) and ambient temperature were retrieved from Weather Underground. Weather data were obtained from the weather stations closest to each sampling site, with an average distance of 21 km (range, 3 to 50 km). For reclaimed water sites, cloud cover, wastewater influent source, and type of primary, secondary, and tertiary treatments were recorded.

### Sample processing.

First 100 ml of universal preenrichment broth (UPB; Accumedia, Lansing, MI, USA) was added to the MMS contained in Whirl-Pak bags and hand-massaged for 1 min. All samples were incubated at 37°C for 18 to 24 h. After incubation, sample bags were again hand-massaged for 1 min. Then, 40 ml of enrichment sample was transferred to a sterile 50-ml labeled conical tube for retention and microbial analysis.

### S. enterica isolation and DNA extraction.

For S. enterica isolation, standard culture methods were used (22). First, 1 ml of the UPB-enriched sample was transferred to 9 ml of tetrathionate (TT; Accumedia, Lansing, MI, USA) broth, and 100 μl was transferred to 10 ml of Rappaport-Vassiliadis (RV; Accumedia) broth for secondary enrichment. Secondary enrichment samples were incubated at 42°C for 18 to 24 h. Next, 10 μl of each broth was streaked onto xylose lysine tergitol-4 (XLT4; Fisher Scientific, Washington, DC, USA) agar and incubated at 42°C for 18 to 24 h. Three presumptive black S. enterica isolates per dilution were transferred to a new XLT4 plate and incubated at 42°C for 18 to 24 h for isolation. DNA extraction of these isolates was done using the InstaGene matrix DNA kit (Bio-Rad, Philadelphia, PA, USA) following the manufacturer’s instructions with the following modifications: instead of suspension in water and pelleting by centrifugation, a single colony was transferred directly to 200 μl of InstaGene matrix in a 1.5-ml microcentrifuge tube. This was a time-saving measure, and DNA quality and yield were found to be acceptable for PCR. Extracted DNA was stored at −20°C until ready for real-time PCR analysis. Additionally, isolates were grown overnight in 1 ml of tryptic soy broth (TSB) supplemented with 15% glycerol (vol/vol) and stored at −80°C for retention.

### L. monocytogenes isolation and DNA extraction.

For L. monocytogenes isolation, 1 ml of the UPB enrichment was transferred to 9 ml of buffered *Listeria* enrichment broth (BLEB; Accumedia, Lansing, MI, USA) and incubated at 37°C for 18 to 24 h. Then, 10 μl of the enriched broth was streaked onto RAPID’L.mono (Bio-Rad, Hercules, CA, USA) agar and incubated at 37°C for 48 h. Three presumptive turquoise L. monocytogenes isolates per dilution were transferred to a new RAPID’L.mono plate and incubated at 37°C for 18 to 24 h for isolation. DNA was extracted from these isolates using the InstaGene matrix DNA kit (Bio-Rad, Philadelphia, PA, USA) as described previously. Extracted DNA was stored at −20°C until ready for real-time PCR confirmation. Isolates were archived by overnight culture in 1 ml of TSB supplemented with 15% glycerol (vol/vol) and 0.6% yeast extract (wt/vol) and stored at −80°C.

### S. enterica and L. monocytogenes quantification.

PCR-confirmed S. enterica and L. monocytogenes colonies from water samples were considered positive for MPN analysis, and pathogen levels were quantified using the method previously described by Sharma et al. (22). Most probable number (MPN) values based on the three volumes (0.1 liters, 1 liter, and 10 liters) with 3 replications per volume were determined using free MPN calculator software (MPN Calculator; Mike Curiale, build 23, VB6) (22). For both S. enterica and L. monocytogenes analyses, the lower limit of detection (LOD) was 0.03 MPN/liter and the upper LOD was 11 MPN/liter. If values were below the LOD, a value of 0.015 MPN/liter (LOD/2) was used, and if values were greater than the upper LOD, a value of 11 MPN/liter was used.

### Real-time PCR assay.

Real-time PCR was performed on presumptive S. enterica and L. monocytogenes isolate DNA using the method previously described by Kawasaki et al. (39). A multiplex real-time PCR assay for S. enterica and L. monocytogenes was conducted on a CFX96 Touch real-time PCR system (Bio-Rad, Philadelphia, USA) using the SensiFAST probe Lo-ROX kit (Meridian Bioscience, Memphis, TN, USA). PCR cycling parameters included an initial denaturation of 10 min at 95°C, followed by 40 cycles of 20 s at 95°C, 30 s at 64°C, and 30 s at 72°C, and a final extension of 7 min at 72°C. Primer and probe (Sigma-Aldrich, St. Louis, MO, USA) sequences used were as previously described (39).

### Statistical analysis.

Statistical analysis was carried out using methods similar to those described in a previous study (22). MPN/liter values were log-transformed prior to using PROC GLM to perform pairwise Tukey’s tests to compare differences in S. enterica and L. monocytogenes between different sampling sites, seasons, and water types. Due to the values from sites MA01 and MA02 (reclaimed water) being at or below the LOD, Tukey’s analysis results were performed both with and without these sites. A logistic regression using PROC LOGISTIC was performed to determine the effect of volume filtration on recovery of S. enterica and L. monocytogenes. Only observations where either S. enterica or L. monocytogenes were recovered were included in the analysis. Statistical analyses were performed using SAS Studio version 9.4 (Carey, NC, USA). A redundancy analysis (RDA) was performed to determine the relationship between water physicochemical factors (explanatory variables) and levels of S. enterica and L. monocytogenes (response variables) to determine the constrained and unconstrained variance (40, 41). Limitations of the statistical analysis include decreased validity and power due to the small and unequal sample sizes.

### Data availability.

The data sets generated for this study are available upon request from the corresponding authors.
